# Self-reported understanding and attitudes of healthcare workers and caregivers toward artificial intelligence in pediatric healthcare in a children’s hospital in China: a cross-sectional study

**DOI:** 10.3389/fpubh.2026.1951776

**Published:** 2026-10-14

**Authors:** Si-Yu Chen, Li Yang, Qi-Gang Ding, Dong-Xin Huang, Qiu-Yu Tang, Xue Kong

**Affiliations:** Fujian Children’s Hospital (Fujian Branch of Shanghai Children’s Medical Center), College of Clinical Medicine for Obstetrics & Gynecology and Pediatrics, Fujian Medical University, Fuzhou, China

**Keywords:** artificial intelligence, caregivers, cross-sectional study, healthcare workers, pediatric healthcare

## Abstract

**Objective:**

To investigate the self-reported understanding and attitudes of healthcare workers and caregivers toward the application of artificial intelligence (AI) in pediatric healthcare in a children’s specialty hospital, and to compare differences between the two groups.

**Methods:**

This single-center cross-sectional questionnaire survey was conducted at Fujian Children’s Hospital from January 2026 to June 2026. A self-designed questionnaire was used to assess healthcare workers’ and caregivers’ self-reported understanding, trust, risk perception, and willingness to accept AI applications in pediatric healthcare. Respondents were divided into groups according to their identity. Categorical variables were compared using the chi-square test or Fisher’s exact test, while continuous variables were compared using the independent-samples *t* test or the Mann–Whitney *U* test.

**Results:**

A total of 261 respondents were included, comprising 159 caregivers and 102 healthcare workers. Healthcare workers had a higher level of self-reported understanding and a higher frequency of use of AI applications in pediatric healthcare than caregivers (*p* < 0.01); however, they also showed stronger perceptions of data security risks (*p* < 0.001). Caregivers reported greater trust in AI-assisted medical decision-making than healthcare workers (*p* < 0.001). Most respondents believed that AI could not replace pediatric healthcare workers, with no statistically significant difference between the two groups (*p* = 0.166). Regarding potential advantages, healthcare workers were more likely to agree that AI could reduce human error (*p* < 0.001). Regarding potential limitations, healthcare workers expressed greater concern about the risks of misdiagnosis, lack of humanistic care, and data privacy and security issues (*p* < 0.001).

**Conclusion:**

In this single-center children’s specialty hospital, healthcare workers reported greater self-rated understanding and more frequent use of AI, together with stronger awareness of several potential risks, whereas caregivers showed greater trust in AI-assisted medical decision-making despite lower self-rated understanding. These findings should be interpreted cautiously and require confirmation in larger multicenter studies before being generalized to broader populations in China.

## Introduction

Artificial intelligence (AI)-related technologies have developed rapidly in the field of medicine in recent years and have gradually been applied across multiple aspects of healthcare, including disease screening, assisted diagnosis, image recognition, clinical decision support, health management, and patient education (1, 2). Previous studies have shown that AI has potential value in improving the efficiency of healthcare services, optimizing resource allocation, and enhancing patients’ quality of life. However, its application in medical settings is also accompanied by concerns regarding safety, effectiveness, privacy protection, accountability, and medical ethics (3, 4). In the field of pediatric healthcare in particular, the application of AI involves not only disease diagnosis and treatment, but also child growth and development monitoring, chronic disease management, family health education, and physician–patient communication (5). Compared with adult healthcare, pediatric healthcare has certain distinctive characteristics. Children may have difficulty clearly describing their symptoms, and their clinical signs and physiological characteristics differ from those of adults. Medical decision-making therefore often requires the joint participation of healthcare workers and caregivers (6).

Healthcare workers, as users and interpreters of AI-based medical tools, may directly influence the extent to which AI technologies are integrated into clinical workflows. Meanwhile, caregivers, as important participants in pediatric medical decision-making, may also affect the practical effectiveness of AI applications in pediatric healthcare through their understanding, trust, and acceptance of AI (7, 8). At present, research on the application of AI in healthcare is increasing; however, most studies have focused on adult patients, general hospitals, or single healthcare workers. Studies examining differences in attitudes between healthcare workers and caregivers in the setting of children’s specialty hospitals remain relatively limited. In pediatric medical settings, healthcare workers and caregivers differ considerably in terms of knowledge background, information needs, and decision-making roles, which may lead to different characteristics in their understanding levels, willingness to accept AI, degree of trust, and ethical concerns regarding AI applications in healthcare. Clarifying these differences may provide evidence for children’s healthcare institutions to develop AI application strategies, optimize health education approaches, improve the quality of physician–patient communication, and strengthen ethical management. On this basis, the present study aimed to conduct a cross-sectional questionnaire survey among healthcare workers and caregivers of pediatric patients at Fujian Children’s Hospital to investigate their self-reported understanding and attitudes toward the application of artificial intelligence in pediatric healthcare and to compare differences between different groups.

## Methods

This was a cross-sectional study conducted at a provincial children’s specialty hospital in China. The study was carried out at Fujian Children’s Hospital from January 2026 to June 2026. The study population consisted of healthcare workers at the children’s specialty hospital and caregivers of pediatric patients receiving medical care. According to participant identity, the study population was divided into two groups: healthcare workers were included in the Healthcare worker group, and primary caregivers of pediatric patients were included in the Caregiver group. This study was approved by the Ethics Committee of Fujian Children’s Hospital. All procedures were conducted in accordance with the Declaration of Helsinki. As this was an anonymous questionnaire-based cross-sectional study and no personally identifiable information was collected, the requirement for written informed consent was waived by the Ethics Committee. Before participation, individuals were provided with information regarding the study purpose, voluntary participation, anonymity, and confidentiality. Voluntary completion and submission of the questionnaire were considered to constitute implied consent to participate in accordance with the approved protocol.

The inclusion criteria for healthcare workers were as follows: 1. being employed at Fujian Children’s Hospital during the study period; 2. voluntary participation in the study; and 3. ability to complete the questionnaire independently. The exclusion criteria were as follows: 1. resignation, completion of further training, or long-term absence from work during the study period; and 2. obviously incomplete questionnaire responses.

The inclusion criteria for caregivers were as follows: 1. being the primary caregiver of a pediatric patient who received medical care at Fujian Children’s Hospital during the study period (For simplicity, primary caregivers are referred to as “caregivers” throughout the manuscript.); 2. age ≥18 years; 3. ability to understand the questionnaire content and complete it independently; and 4. voluntary participation in the survey. The exclusion criteria were as follows: 1. inability to provide valid survey feedback; 2. incomplete questionnaire responses; and 3. not being the primary caregiver of a pediatric patient.

During the study period, questionnaires were distributed by the researchers to healthcare workers and caregivers who met the inclusion criteria. To improve sample coverage, data were collected using a combination of electronic and paper questionnaires. Electronic questionnaires were distributed via QR codes or web links, while paper questionnaires were distributed and collected on site by uniformly trained researchers. The electronic and paper versions of the questionnaire were identical in item wording, response options, and scoring, and differed only in the mode of administration. All questionnaires were completed anonymously. Questionnaire administration mode was not recorded for individual participants; therefore, potential mode effects could not be assessed. In the present study, “AI” primarily referred to generative AI-based conversational tools capable of responding to users’ questions and generating text-based information, such as ChatGPT, DeepSeek, Kimi, and Doubao. The questionnaire focused mainly on participants’ perceptions and use of these general-purpose AI conversational tools in the context of pediatric healthcare, rather than on specialized clinical AI systems such as medical imaging algorithms or dedicated clinical decision-support systems.

The questionnaire was developed based on a review of previous studies on AI-related perceptions and applications in healthcare (9, 10). The items covered self-reported understanding, AI usage, perceived benefits and risks, trust, ethical concerns, and willingness to use AI. The questionnaire was reviewed by pediatric healthcare clinicians, and a pilot test was conducted among 20 participants (10 healthcare workers and 10 primary caregivers) to assess clarity and comprehensibility before formal administration. The questionnaire mainly included the following aspects: general demographic characteristics, including age, sex, and educational level; self-reported understanding of AI technology and its medical applications; attitudes toward and acceptance of AI applications in pediatric healthcare; trust in AI medical applications; and concerns regarding potential risks and ethical issues. Most questionnaire items used a 4-point ordered response scale scored from 1 to 4, with higher scores indicating a greater level of the construct assessed. The AI-understanding item reflected self-reported understanding rather than objective AI knowledge and was scored from 1 (“no understanding”) to 4 (“very good understanding”). No composite or domain scores were calculated, and each item was analyzed separately. The AI-replacement item was analyzed as a binary variable, while perceived advantages and disadvantages were assessed using multiple-response items (Supplementary material 1). Before questionnaire distribution, investigators received standardized training regarding the inclusion and exclusion criteria and questionnaire completion instructions. All data were entered into the database after verification by two researchers.

### Statistical analysis

Sample size estimation was based on the comparison of AI-related perception scores between healthcare workers and caregivers. Assuming a two-sided *α* of 0.05, 80% statistical power, and a moderate standardized effect size (Cohen’s *d* = 0.50), a minimum of 90 participants per group was required. Allowing for approximately 10% invalid or incomplete questionnaires, the target sample size was increased to approximately 200 participants in total.

Continuous score-based variables were tested for normality using the Shapiro–Wilk test. All eight score-based variables reported in Table 1, including self-reported understanding of AI applications in pediatric healthcare, frequency of AI software use, perceived accuracy of AI-provided information, perceived potential of AI to improve the quality of pediatric care, perceived potential of AI to reduce healthcare costs, trust in AI involvement in medical decision-making, perceived data security risks of AI use, and willingness to use AI in future healthcare encounters, were approximately normally distributed and are presented as the mean ± standard deviation. Effect sizes with 95% CIs were reported as mean differences for score-based outcomes and ORs for categorical outcomes. Comparisons between the two groups for these variables were performed using the independent-samples t test. Categorical variables were expressed as frequencies and percentages and compared using the chi-square test or Fisher’s exact test, as appropriate. Following inspection of the initial between-group comparisons, four score-based outcomes showing statistically significant differences: self-reported AI understanding, frequency of AI use, trust in AI-assisted decision-making, and perceived data security risks, were selected *post hoc* for additional multivariable linear regression analyses. These exploratory models assessed associations with participant group after adjustment for age group, sex, and educational level; the outcomes were not prespecified for adjusted analysis. *Post hoc* exploratory linear regression analyses in Table 2 examined associations of self-reported AI understanding with trust, perceived data security risks, and willingness to use AI, adjusting for participant group, age group, sex, and educational level as categorical covariates. A *p* value <0.05 was generally considered statistically significant. To account for multiple testing of the questionnaire items reported in Tables 1, 3, a Bonferroni correction was applied across the 19 comparisons, resulting in a corrected significance threshold of *p* < 0.0026 (0.05/19). Statistical analyses were performed using SPSS version 25 (IBM Corp., Armonk, NY, USA).

**Table 1:** Comparison of self-reported understanding and attitudes toward AI applications in pediatric healthcare.

Item	Caregiver group (*n* = 159)	Healthcare worker group (*n* = 102)	*p* value^*^	Effect estimate (95% CI)
Understanding of AI applications in pediatric healthcare	2.41 ± 0.71	2.71 ± 0.62	<0.001	MD 0.29 (0.13–0.45)
Frequency of AI software use	2.38 ± 0.59	2.61 ± 0.53	0.002	MD 0.22 (0.09–0.36)
Perceived accuracy of AI-provided information in pediatric healthcare	2.82 ± 0.61	2.93 ± 0.65	0.183	MD 0.11 (−0.05 to 0.27)
Perceived potential of AI to improve the quality of pediatric care	2.97 ± 0.72	2.88 ± 0.71	0.307	MD −0.09 (−0.27 to 0.09)
Perceived potential of AI to reduce healthcare costs	2.94 ± 0.64	2.82 ± 0.60	0.150	MD −0.11 (−0.27 to 0.04)
Trust in AI involvement in medical decision-making	2.81 ± 0.67	2.49 ± 0.54	<0.001	MD −0.31 (−0.46 to −0.17)
Whether AI can replace pediatric healthcare professionals, no (%)	132 (83.0)	91 (89.2)	0.166	OR 1.69 (0.80–3.58)
Perceived data security risks of AI use	2.25 ± 0.48	2.58 ± 0.52	<0.001	MD 0.33 (0.20–0.45)
Willingness to use AI in future healthcare encounters	2.94 ± 0.76	2.86 ± 0.63	0.352	MD −0.08 (−0.25 to 0.09)

MD, mean difference; OR, odds ratio. *p* values shown are unadjusted. For the 19 questionnaire comparisons across Tables 1, 3, the Bonferroni-corrected significance threshold was *p* < 0.0026.

**Table 2:** Exploratory associations of self-reported AI understanding with trust, perceived data security risks, and willingness to use AI.

Item	Adjusted *β* (95% CI)	*p* value
Trust in AI involvement in medical decision-making	−0.002 (−0.115 to 0.111)	0.971
Perceived data security risks of AI use	0.007 (−0.082 to 0.097)	0.870
Willingness to use AI in future healthcare encounters	−0.059 (−0.189 to 0.071)	0.372

**Table 3:** Comparison of perceived advantages and disadvantages of AI between healthcare workers and caregivers.

Item	Caregiver group (*n* = 159)	Healthcare worker group (*n* = 102)	*p* value^*^	OR (95% CI)
Perceived advantages, (%)				
Convenience of use	131 (82.4)	78 (76.5)	0.243	0.69 (0.38–1.28)
Alleviating shortages of healthcare resources	100 (62.9)	75 (73.5)	0.074	1.64 (0.95–2.83)
Improving diagnostic efficiency	95 (59.7)	70 (68.6)	0.147	1.47 (0.87–2.49)
Reducing human errors	89 (56.0)	80(78.4)	<0.001	2.86 (1.62–5.04)
Improving treatment outcomes	66 (41.5)	39 (38.2)	0.599	0.87 (0.52–1.45)
Perceived disadvantages, (%)				
Insufficient accuracy and potential misdiagnosis	91 (57.2)	82 (80.4)	<0.001	3.06 (1.71–5.48)
Lack of humanistic care	56 (35.2)	77 (75.5)	<0.001	5.67 (3.25–9.88)
Data privacy and security concerns	68(42.8)	84 (82.4)	<0.001	6.25 (3.43–11.36)
Unclear responsibility in the event of misdiagnosis	95 (59.7)	68 (66.7)	0.260	1.35 (0.80–2.27)
Potential decline in physicians’ clinical judgment due to reliance on AI	74 (46.5)	60 (58.8)	0.053	1.64 (0.99–2.71)

Participants were allowed to select multiple response options. *p* values shown are unadjusted. For the 19 questionnaire comparisons across Tables 1, 3, the Bonferroni-corrected significance threshold was *p* < 0.0026.

## Results

A total of 304 eligible participants were identified, of whom 292 were approached and 268 returned questionnaires. After excluding 7 incomplete questionnaires, 261 participants were included in the final analysis (Figure 1). Among the 102 healthcare workers, 59 (57.8%) were physicians and 43 (42.2%) were nurses. There was no statistically significant difference in age distribution between the two groups, with most participants concentrated in the 30–39-year and 40–49-year age ranges. The sex distribution also did not differ significantly between the two groups (*p* > 0.05). In terms of educational level, the proportion of participants with a bachelor’s degree or above was significantly higher in the Healthcare worker group than in the Caregiver group, whereas the proportion of participants with lower educational levels was higher in the Caregiver group, indicating a significant difference in educational background between the two groups (Table 4).

**Figure 1:**
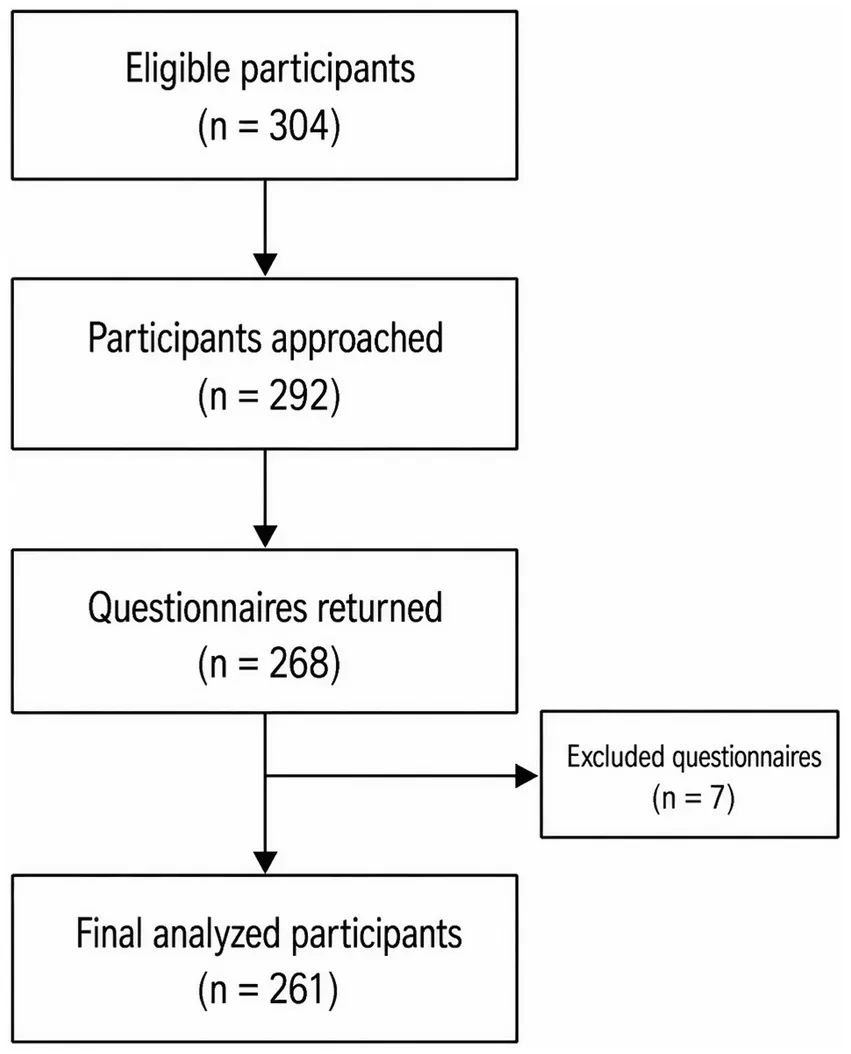
Participant flow diagram.

**Table 4:** Baseline characteristics of the participants.

Item	Caregiver group (*n* = 159)	Healthcare group (*n* = 102)	*p* value
Age, (%)			
20–29 years	22 (13.8)	12 (11.8)	0.407
30–39 years	67 (42.1)	53 (52.0)	
40–49 years	48 (30.2)	27 (26.5)	
50–59 years	19 (11.9)	10 (9.8)	
≥60 years	3 (1.9)	0 (0.0)	
Sex, (%)			
Women	104 (65.4)	57 (55.9)	0.122
Men	55 (34.6)	45 (44.1)	
Educational level, (%)			
Below senior high school	10 (6.3)	0 (0.0)	–
High school	32 (20.1)	0 (0.0)	
Junior college	71 (44.7)	28 (27.5)	
Bachelor degree or above	46 (28.9)	74 (72.5)	

Regarding self-reported understanding and attitudes toward the application of AI in pediatric healthcare, healthcare workers had a significantly higher level of understanding of AI applications than caregivers (2.71 ± 0.62 vs. 2.41 ± 0.71, *p* < 0.001). They also reported a higher frequency of using AI software when encountering pediatric healthcare-related issues (2.61 ± 0.53 vs. 2.38 ± 0.59, *p* = 0.002). The Caregiver group showed significantly greater trust in AI involvement in medical decision-making than the Healthcare worker group (2.81 ± 0.67 vs. 2.49 ± 0.54, *p* < 0.001). Healthcare workers perceived significantly greater data security risks associated with AI use than caregivers (2.58 ± 0.52 vs. 2.25 ± 0.48, *p* < 0.001). No statistically significant differences were observed between the two groups in perceived accuracy of information provided by AI, the potential of AI to improve healthcare quality, the potential of AI to reduce medical costs, whether AI could replace pediatric healthcare workers, or willingness to use AI in the future (all *p* > 0.05). All differences that were statistically significant in the initial analyses remained significant after Bonferroni correction for multiple comparisons (Table 1).

In the comparison of perceived potential advantages and limitations of AI, healthcare workers were significantly more likely than caregivers to select “reducing human error” as a potential advantage (78.4% vs. 56.0%, *p* < 0.001). No statistically significant differences were found between the two groups in the perceived advantages of “convenient use,” “alleviating shortages of medical resources,” “improving diagnostic efficiency,” or “improving treatment outcomes” (all *p* > 0.05) (Table 3). Regarding the potential limitations of AI, healthcare workers showed higher levels of concern than caregivers about “insufficient accuracy and possible misdiagnosis” (80.4% vs. 57.2%, *p* < 0.001), “lack of humanistic care” (75.5% vs. 35.2%, *p* < 0.001), and “data privacy and security issues” (82.4% vs. 42.8%, *p* < 0.001). However, there were no statistically significant differences between the two groups in concerns regarding “unclear responsibility for misdiagnosis” (66.7% vs. 59.7%, *p* = 0.260) or “reliance on AI potentially leading to a decline in physicians’ clinical judgment” (58.8% vs. 46.5%, *p* = 0.053) (Table 3).

In the *post hoc* exploratory models, the adjusted coefficients for healthcare worker status were 0.315 for self-reported AI understanding, 0.218 for frequency of AI use, −0.253 for trust in AI-assisted decision-making, and 0.374 for perceived data security risks (Table 5). In the additional exploratory analyses, self-reported AI understanding was not statistically significantly associated with trust in AI involvement in medical decision-making (adjusted *β* = −0.002, 95% CI: −0.115 to 0.111; *p* = 0.971), perceived data security risks (adjusted *β* = 0.007, 95% CI: −0.082 to 0.097; *p* = 0.870), or willingness to use AI in future healthcare encounters (adjusted *β* = −0.059, 95% CI: −0.189 to 0.071; *p* = 0.372), after adjustment for participant group, age group, sex, and educational level (Table 2).

**Table 5:** *Post hoc* exploratory multivariable linear regression analyses of selected AI-related outcomes by participant group.

Item	Adjusted *β* (95% CI)	*p* value
Self-reported understanding of AI applications	0.315 (0.121–0.508)	0.002
Frequency of AI software use	0.218 (0.058–0.378)	0.008
Trust in AI-assisted decision-making	−0.253 (−0.428 to −0.078)	0.005
Perceived data security risks	0.374 (0.235–0.514)	<0.001

Adjusted for educational level, age, and sex; caregivers were used as the reference group. *β* values are unstandardized regression coefficients for healthcare worker status relative to caregiver status. Outcomes were selected after inspection of the initial between-group comparisons. *p* values and confidence intervals were not adjusted for multiple testing.

## Discussion

This study focused on two key stakeholder groups in a children’s specialty hospital, namely healthcare workers and caregivers of pediatric patients, and compared their self-reported understanding, trust, risk perception, and willingness to accept AI applications in pediatric healthcare. The results showed that both groups generally held relatively positive attitudes toward AI applications; however, the structure of their attitudes was not entirely consistent. Healthcare workers had greater understanding of AI and used AI more frequently, while they were also more sensitive to issues such as data security, risk of misdiagnosis, and lack of humanistic care. In contrast, caregivers showed greater trust in AI involvement in medical decision-making. These observed differences may reflect variations in participants’ professional backgrounds, information needs, risk perceptions, and roles in healthcare decision-making (11–13). For the four outcomes selected for *post hoc* regression analysis, the directions of the group differences were unchanged after adjustment for age group, sex, and educational level. However, because these outcomes were selected after inspection of the initial comparisons, the adjusted findings should be interpreted as exploratory rather than as independent confirmation of the observed differences.

This study found that healthcare workers had a higher level of self-reported understanding and a higher frequency of use of AI applications in pediatric healthcare than caregivers. One possible explanation for these differences is that healthcare workers may have greater exposure to AI-related information and applications through their professional and clinical environments. Their greater concern regarding data security, misdiagnosis, and humanistic care may also reflect greater awareness of clinical uncertainty and the limitations of AI. However, these explanations were not directly examined in the present study and should therefore be considered hypotheses rather than established mechanisms. Previous surveys among healthcare workers in China have also shown that medical staff generally hold positive attitudes toward AI, but their understanding of basic AI understanding, application standards, and ethical risks remains insufficient (14, 15). Although healthcare workers reported greater self-reported AI understanding, lower trust, and higher perceived data security risks than caregivers, these group-level differences do not establish an individual-level association between understanding and attitudes. In the additional exploratory analyses, self-reported AI understanding was not statistically significantly associated with trust, perceived data security risks, or willingness to use AI after adjustment for participant group and demographic characteristics. These findings do not establish the absence of an association, but they do not support attributing the observed differences in attitudes to self-reported understanding alone.

Compared with healthcare workers, caregivers showed greater trust in AI involvement in medical decision-making. One possible explanation is that caregivers may value the rapid, convenient, and accessible health information provided by conversational AI tools (16). Such characteristics could contribute to greater receptiveness toward AI-assisted health information. However, this interpretation was not directly tested in the present study and should be regarded as a possible explanation rather than a causal mechanism. Previous studies on parents’ attitudes toward AI technologies in pediatrics have also shown that parents are generally open to AI-assisted pediatric healthcare, while also expressing concerns about information accuracy, privacy protection, shared decision-making, medical equity, and costs (17, 18). In this study, caregivers’ relatively high level of trust in AI also indicates that AI has a certain application basis in pediatric health consultation, pre-visit information acquisition, and health education.

Given the potential risks associated with AI-generated health information, clear guidance should be provided to caregivers, emphasizing that AI-generated information cannot replace physicians’ diagnosis and treatment, particularly physical examination, dynamic assessment of disease progression, and individualized medication decisions. It should also be noted that AI applications in healthcare are heterogeneous and may involve different benefits and risks depending on their intended use. The present study primarily focused on generative AI-based conversational tools used for health-related information and interaction. Therefore, the findings should not be directly extrapolated to specialized applications such as medical imaging algorithms or dedicated clinical decision-support systems. Even among conversational AI tools, perceptions may differ according to whether they are used for health education, preliminary information seeking, or decision support.

Regarding the potential limitations of AI, healthcare workers expressed significantly greater concern than caregivers about the risk of misdiagnosis, lack of humanistic care, and data privacy and security issues. This finding is consistent with the main concerns highlighted in current ethical research on medical AI. Previous systematic reviews and international guidelines have emphasized that medical AI should focus on issues such as patient autonomy, privacy protection, algorithmic bias, accountability, transparency, explainability, equity, and regulatory mechanisms (19). Children are a particularly vulnerable population in the context of medical AI applications. On the one hand, pediatric medical data involve long-term health trajectories, and data leakage or inappropriate use may have more persistent consequences. On the other hand, children are usually unable to make medical decisions completely independently, and the application of AI also involves ethical issues such as guardian consent, children’s informed participation, and family-based shared decision-making. Most respondents believed that AI could not completely replace pediatric healthcare workers. Previous studies on trust in and acceptance of medical AI have suggested that transparency, physician involvement, clear boundaries of responsibility, and patients’ understanding of the system are important factors influencing AI acceptance (20, 21). Therefore, AI in future pediatric healthcare should be positioned as an “assistive tool” rather than a “replacement agent.” Its application model should emphasize physician leadership, AI assistance, caregiver awareness, and shared decision-making.

These findings may have practical implications for AI literacy and communication strategies in pediatric healthcare. Educational approaches should be tailored to different stakeholder groups, with greater emphasis on the appropriate use, limitations, and risks of AI for caregivers, while supporting healthcare workers in developing a balanced understanding of both the potential benefits and limitations of AI.

This study has several limitations. First, this was a single-center cross-sectional survey conducted in one children’s specialty hospital, and voluntary participation may have introduced selection bias; therefore, the generalizability of the findings is limited. Second, although the questionnaire was developed with reference to relevant literature and pilot-tested before formal administration, it remains a self-developed instrument without formal psychometric validation. In addition, AI self-reported understanding was assessed based on self-reported understanding rather than an objective knowledge test. Third, although multivariable analyses were performed to adjust for differences in educational level, age, and sex, residual confounding from unmeasured factors cannot be excluded. Fourth, the study did not further examine differences across professional roles, clinical departments, patient disease types, or specific AI application scenarios. The absence of participant-level information on questionnaire administration mode prevented assessment of potential response differences between electronic and paper questionnaires. Finally, the cross-sectional design precludes causal inference, and self-reported responses may be subject to response bias, including social desirability bias.

The outcomes for the adjusted group comparisons were selected after inspection of the initial results. This data-dependent selection and the use of nominal regression inference may yield overly optimistic findings; these analyses should therefore be regarded as exploratory and require confirmation in an independent sample.

## Conclusion

In this single-center children’s specialty hospital, healthcare workers reported greater self-rated understanding and more frequent use of AI, together with stronger awareness of several potential risks, whereas caregivers showed greater trust in AI-assisted medical decision-making despite lower self-rated understanding. These findings should be interpreted cautiously and require confirmation in larger multicenter studies before being generalized to broader populations in China.

## Data Availability

The raw data supporting the conclusions of this article will be made available by the authors, without undue reservation.
