# Supplementary material for: Self-reported understanding and attitudes of healthcare workers and caregivers toward artificial intelligence in pediatric healthcare in a children’s hospital in China: a cross-sectional study
Source: Front Public Health. 2026 Oct 14;14:1951776. doi: 10.3389/fpubh.2026.1951776 (PMC13713840; doi:10.3389/fpubh.2026.1951776)
Supplement: Supplementary file 1 [file Supplementary_file_1.docx]

**English Version of the Questionnaire Survey of Healthcare Workers’ and Caregivers’ Attitudes Toward Artificial Intelligence Applications in Pediatric Healthcare**

I. Basic Information

1. What is your identity?

A. Healthcare worker

B. Primary caregiver of a pediatric patient

2. What is your age?

A. 20–29 years

B. 30–39 years

C. 40–49 years

D. 50–59 years

E. 60 years or older

3. What is your sex?

A. Male

B. Female

4. What is your highest educational level?

A. Junior high school or below

B. Senior high school / technical secondary school

C. College / bachelor’s degree

D. Master’s degree or above

II. Self-Reported Understanding and Attitudes Toward Artificial Intelligence

5. Have you ever used artificial intelligence (AI)-related technologies or software in your daily clinical work or daily life to obtain information about child health?

A. Yes

B. No

**Operational definition of AI**

In this study, “AI” primarily referred to generative AI-based conversational tools capable of responding to users’ questions and generating text-based information, such as ChatGPT, DeepSeek, Kimi, and Doubao.

6. How would you rate your understanding of AI applications in pediatric healthcare?

A. No understanding at all

B. Limited understanding

C. Moderate understanding

D. Very good understanding

7. When you encounter pediatric healthcare-related questions, how often do you use AI software?

A. Never

B. Occasionally

C. Often

D. Always

8. How accurate do you think the information provided by AI software in pediatric healthcare is?

A. Inaccurate

B. Somewhat accurate

C. Mostly accurate

D. Very accurate

9. To what extent do you think AI can help improve the quality of pediatric care?

A. Not at all

B. To a limited extent

C. To a moderate extent

D. To a great extent

10. Do you think AI has the potential to reduce healthcare costs?

A. No potential at all

B. Some potential

C. Substantial potential

D. Very substantial potential

III. Trust and Ethical Concerns

11. How much do you trust AI involvement in medical decision-making?

A. Do not trust at all

B. Do not trust very much

C. Moderately trust

D. Trust very much

12. What do you consider to be the potential advantages of AI in pediatric healthcare? (Multiple responses allowed)

A. Convenience of use

B. Alleviating shortages of healthcare resources

C. Improving diagnostic efficiency

D. Reducing human errors

E. Improving treatment outcomes

F. None

13. What do you consider to be the potential disadvantages of AI in pediatric healthcare? (Multiple responses allowed)

A. Insufficient accuracy and potential misdiagnosis

B. Lack of humanistic care

C. Data privacy and security concerns

D. Unclear responsibility in the event of misdiagnosis

E. Potential decline in physicians’ clinical judgment due to reliance on AI

F. None

14. Do you think AI can replace pediatric healthcare workers in pediatric healthcare?

A. Yes

B. No

15. Do you think the use of AI in pediatric healthcare increases risks related to patient data security?

A. Not at all

B. To a limited extent

C. To some extent

D. To a great extent

16. Would you be willing to have greater exposure to or use of AI in future healthcare encounters?

A. Completely unwilling

B. Somewhat unwilling

C. Neutral

D. Very willing

**Scoring Note**

**•** Questions 6,7,9, 10, 11, 15, and 16 were coded from 1 to 4 in ascending order from option A to option D, such that higher scores reflected greater self-reported understanding, more frequent AI use, stronger perceived benefit, greater trust, greater perceived data-security risk, or greater willingness to use AI, as applicable.

**•** Questions 5 and 14 were binary single-choice items. Questions 12 and 13were multiple-response items; each option was analyzed separately as selected/not selected.
